# Molecular Profiling in Non-Small-Cell Lung Cancer: A Single-Center Study on Prevalence and Prognosis

**DOI:** 10.3390/curroncol32050274

**Published:** 2025-05-09

**Authors:** Mustafa Özgür Arıcı, Bora Demirkan, Ebru Taştekin, Derya Kıvrak Salim

**Affiliations:** 1 Department of Medical Oncology, Antalya Training and Research Hospital, 07100 Antalya, Türkiye; mustafaozgur.arici@saglik.gov.tr; 2 Medical Oncology, Muş State Hospital, 49200 Muş, Türkiye; 3 Department of Pathology, Trakya University Faculty of Medicine, 22030 Edirne, Türkiye; borademirkan@trakya.edu.tr (B.D.); ebrutastekin@trakya.edu.tr (E.T.)

**Keywords:** non-small-cell lung cancer, molecular profiling, next-generation sequencing, targeted therapy, survival

## Abstract

The aim of this study is to evaluate the prognostic value of molecular profiling in patients with metastatic non-small-cell lung cancer (NSCLC). This single-center study included patients diagnosed and treated between July 2020 and April 2024. The molecular profiles of patients detected by either next-generation sequencing or conventional methods were reviewed retrospectively. Survival analyses were conducted based on the targetable alterations and treatments received. Seventy patients were included, with a median age of 65 years and a median overall survival (OS) of 13 months. Of all patients, 56 (80%) had at least one molecular alteration, and the most frequent alteration was TP53 (52.9%), followed by KRAS (20%) and EGFR (8.6%). Eighteen patients (25.7%) had an alteration amenable to targeted therapy. Patients who could reach a matched targeted therapy at any treatment line exhibited a longer median OS compared to those who could not (not reached vs. 6.9 months, *p* = 0.042). Patients with a targetable alteration for first-line treatment demonstrated a longer progression-free survival compared to those without a targetable alteration (not reached vs. 4.9 months, *p* = 0.006). According to current guidelines, conducting molecular testing to identify all potential targetable alterations in NSCLC is the cornerstone of the treatment decision process. The survival analysis in this study emphasized the impact of the use of targeted therapies on the survival outcomes.

## 1. Introduction

The development of immunotherapy and targeted therapies has significantly improved the treatment of lung cancer [1,2]. Depending on the biomarker, the use of these novel therapies has led to increased survival rates [2]. Therefore, the identification of biomarkers is important to guide treatment and optimize outcomes for patients with non-small-cell lung cancer (NSCLC), especially those with advanced or metastatic disease [1,2]. In accordance with current international guidelines, patients with lung cancer diagnosed with advanced or metastatic non-squamous histology should undergo testing for alterations in epidermal growth factor receptor (EGFR), anaplastic lymphoma kinase (ALK), ROS proto-oncogene 1 (ROS1), V-Raf murine sarcoma viral oncogene homolog B (BRAF), Kirsten rat sarcoma virus (KRAS), neurotrophic tyrosine receptor kinase (NTRK1/2/3), rearranged during transfection proto-oncogene (RET), mesenchymal–epithelial transition proto-oncogene (MET) exon 14 skipping, and erb-b2 receptor tyrosine kinase 2 (ERBB2)/(HER2) [3,4]. All patients with squamous cell histology, not only those with mixed histology or never smokers, are also recommended to have molecular testing since targetable molecular alterations may be found in up to 10% [3].

Molecular profiling of lung cancer has historically involved conventional and single-test techniques, such as fluorescence in situ hybridization (FISH), immunohistochemistry (IHC), and reverse-transcription polymerase chain reaction (RT-PCR) [5]. However, molecular characterization takes longer with these procedures since they require more material for analysis. Next-generation sequencing (NGS) can concurrently analyze a wide variety of genomic changes across multiple genes using a limited sample size in a shorter duration [6]. NGS enhances the probability of detecting targetable alterations by its ability to find small mutations throughout the analyzed gene regions. Nevertheless, NGS has some unique pitfalls, and using NGS alone may not always produce actionable data in some cases [7,8]. By integrating NGS with the data from the abovementioned conventional molecular techniques, the detection rate of the targetable molecular alterations may increase [9,10]. A further limitation of NGS is its lack of accessibility in all cancer clinics around the world [11].

In accordance with global data [12], lung cancer remains the most prevalent diagnosed malignancy and the leading cause of mortality due to cancer in Türkiye [13]. Approximately 85% of lung cancer cases in Türkiye are non-small-cell histology, and more than half of newly diagnosed patients receive an advanced stage diagnosis [13]. Following the initial diagnosis, alterations in EGFR, ALK, and ROS1 using IHC, FISH, or RT-PCR are routinely evaluated in many centers under the reimbursement of the Social Security Institution (SSI) [13]. Comprehensive molecular profiling to identify potentially targetable alterations, especially through NGS, has not become widespread due to cost, availability, and the lack of reimbursement coverage.

Detection of all the abovementioned potential targetable alterations through molecular profiling is critical for optimal treatment decision-making. However, based on real-world evidence, most patients with NSCLC fail to receive molecular testing [11,14]. In this study, we retrospectively analyzed the medical records of patients with metastatic NSCLC to define their molecular profile. Furthermore, we aimed to evaluate the prognostic value of molecular profiling performed by either conventional methods or NGS on survival outcomes.

## 2. Materials and Methods

### 2.1. Study Design and Data Collection

This retrospective observational single-center study was approved by the Institutional Review Board of Antalya Training and Research Hospital (Approval date: 23 May 2024, number: 7/23). Informed consent was waived because of the retrospective nature of the study.

Patients diagnosed and treated with advanced lung cancer between July 2020 and April 2024 were retrospectively reviewed. All patients received a diagnosis of NSCLC histopathologically and underwent molecular analysis. Patients with insufficient archival records or follow-up data were not included in the study. The main data obtained from the archival records included patient demographics, smoking status, the pathological subtype, and the sites of metastasis. The results of the EGFR mutations and ALK or ROS1 rearrangements performed as a reflex test by the local laboratory were also recorded. The date of diagnosis, the treatment lines, response to first-line treatment, and the date of progression or exitus were noted.

### 2.2. Molecular Alterations

EGFR sensitizing mutations or deletions, ALK or ROS1 rearrangements, KRAS G12C mutations, RET fusions, MET exon 14 skipping mutations, HER2 exon 20 mutations, HER2 amplifications, NTRK rearrangements, and BRAF V600E mutations were considered as potential druggable alterations for any treatment line [3]. In addition, alterations with an approved treatment option for the first-line treatment recommended by current international guidelines were noted separately [3,4].

### 2.3. Nucleic Acid Extraction and Next-Generation Sequencing

The QIAamp DNA FFPE Advanced Kit (Qiagen, Hilden, Germany) was used for DNA isolation, and the Qiagen RNeasy FFPE Kit (Qiagen, Hilden, Germany) was used for RNA isolation. Qubit dsDNA HS Assay Kit and Qubit RNA HS Assay Kit (ThermoFisher Scientific, Waltham, MA, USA) were used to assess DNA and RNA concentrations, and measurements were performed on the Qubit 4.0 (Marsiling, Singapore) device. The NGS study was started with a concentration of 10 to 250 µg/µL in 16.75 µL for DNA and 50 to 250 µg/µL in 5 µL for RNA. To generate a DNA library, Qiaseq Custom Lung DNA Kit (333525) (Qiagen, Hilden, Germany) was used, and the procedures of the kit were followed. Qiaseq Lung custom RNAscan Kit (333625) (Qiagen, Hilden, Germany) was used to generate the RNA library, and the procedures of the kit were followed. The size of the library products was measured using the Qiaxcel DNA High Resolution Kit (929002) (Qiagen, Hilden, Germany), and the concentration of the library products was determined by qPCR using the Qiaseq Quant assay (333314) (Qiagen, Hilden, Germany). The prepared libraries were sequenced at 151 × 2 base length on NextSeq (Illumina, San Diego, CA, USA), and platform-specific Qiagen software (9.4.1.20250320) pathways were used for variant calling. The Qiagen Clinical Insight-Interpret program was used for further variant analysis, mutation identification, and clinical evaluation.

### 2.4. Treatments

All patients were treated with drugs recommended by international guidelines that are licensed and accessible in our country. Patients with EGFR, ALK, or ROS1 alterations received erlotinib, alectinib, or crizotinib, respectively. Among the patients without targetable alterations, those who could access immunotherapy were given chemoimmunotherapy for first-line treatment. Among these patients, those with adenocarcinoma histology received pembrolizumab (200 mg) + platinum (cisplatin 75 mg/m^2^ or carboplatin 5 AUC) + pemetrexed (500 mg/m^2^) on day 1 every 21 days, while patients with squamous histology received the combination of pembrolizumab (200 mg) + carboplatin (6 AUC) + paclitaxel (200 mg/m^2^) on day 1 every 21 days. Chemotherapy was given to patients who could not access immunotherapy or for whom immunotherapy was contraindicated. Patients with adenocarcinoma received platinum (cisplatin 75 mg/m^2^ or carboplatin 5 AUC) plus pemetrexed (500 mg/m^2^) on day 1 every 21 days. Patients with squamous cell carcinoma received either carboplatin (6 AUC) with paclitaxel (200 mg/m^2^) on day 1 or platinum (cisplatin 75 mg/m^2^ or carboplatin 5 AUC on day 1) with gemcitabine (1000 mg/m^2^ on days 1 and 8) every 21 days, depending on the physician’s preference. Patients with large cell carcinoma were treated with platinum (cisplatin 75 mg/m^2^ or carboplatin 5 AUC on day 1) plus etoposide (100 mg/m^2^ on days 1 to 3) every 21 days. Considering the existence of side effects, the patient’s performance status, or the laboratory results prior to each cycle, the appropriate dose adjustment or dose delay was made based on the physician’s judgment.

### 2.5. Statistical Analysis

We used SPSS version 25.0 (IBM Corp., Armonk, NY, USA) and the GraphPad Prism 9.0 package for statistical analyses and graph presentation. Continuous variables were presented as medians (interquartile range (IQR) or range), and categorical variables were given as numbers (percentages). Fisher’s exact test was used to assess the statistical association between different variables. Kaplan–Meier survival curves were used for estimating PFS and OS, with differences between them were compared using the log-rank test. Progression-free survival (PFS) was defined as the time from the date of first-line treatment to the date of progression or death from any cause, whichever occurred first, or the last follow-up visit for patients who were alive without progression. Overall survival (OS) was defined as the time from the date of diagnosis of metastatic disease to the date of death or the last follow-up. A *p* value < 0.05 was considered statistically significant.

## 3. Results

### 3.1. Baseline Patient Characteristics

A total of 70 patients were included in the study. Baseline characteristics are summarized in Table 1. The median age was 65 (range, 43–78), and 22.9% of the patients were female. Never smokers constituted 21.4% of the cohort, and 62.9% of the patients had a diagnosis of adenocarcinoma. Thirty-five patients (50%) exhibited negative (<1%) PD-L1 TPS levels.

### 3.2. Molecular Profile of the Patients

**Figure 1:**
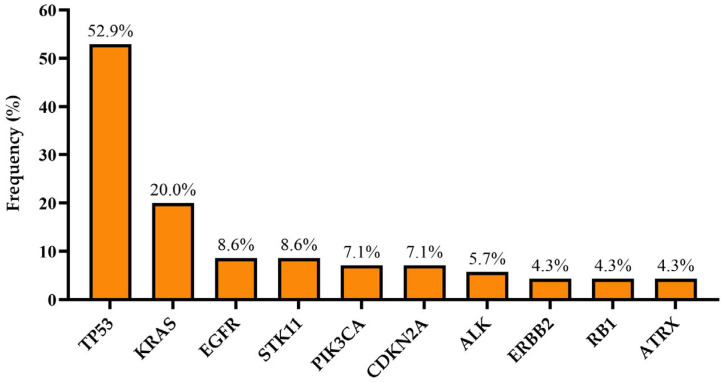
Molecular alterations with a frequency of more than 4% in all cohorts.

Among the 70 patients, 56 (80%) were found to have at least one molecular alteration, and 18 of the patients (25.7%) had an alteration amenable to targeted therapy in any treatment line. Figure 1 shows molecular alterations with a percentage of more than 4%. The most frequent alteration was TP53 (52.9%), followed by KRAS (20%), EGFR (8.6%), and STK11 (8.6%). Alterations with a frequency of less than 4% are listed in Table 2.

### 3.3. Relationships Between Clinicopathological Features and Alterations

The EGFR mutation was significantly higher in females than in males (*p* = 0.002), as well as in never smokers than in patients with a history of smoking (*p* < 0.001). The same findings were observed for ALK (females vs. males; *p* = 0.002 and never smokers vs. smokers; *p* = 0.001). Although alterations in KRAS and ERBB2 were observed at higher percentages in males or those with a history of smoking, there was no statistical association between these characteristics and alterations (*p* > 0.05). We found that female patients were statistically more likely to have a targeted treatment option regardless of smoking history (*p* < 0.001). A similar trend was observed for smoking history, with never smokers having more targeted treatment options than smokers (*p* < 0.001).

Regarding the relationship between specific alterations and metastatic sites, no statistical association was found between alterations in EGFR, ALK, ERBB2, or KRAS and the presence of either brain, liver, or bone metastases (*p* > 0.05).

### 3.4. Targetable Alterations

**Figure 2:**
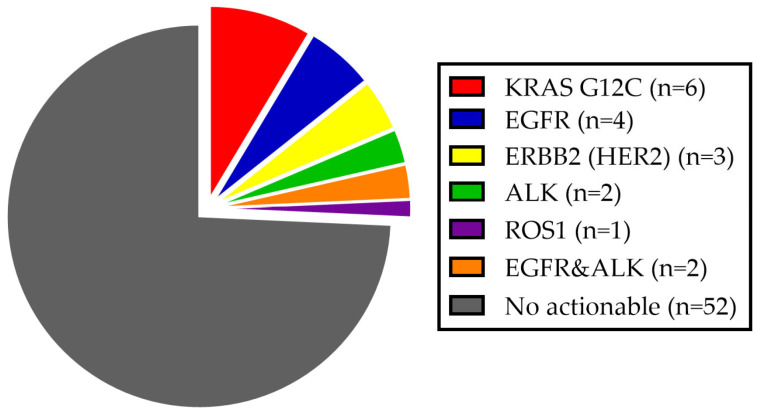
Targetable molecular alterations in our cohort according to current international guidelines.

An overview of targetable molecular alterations is presented in Figure 2. Among the 14 KRAS alterations, 6 (42.8%) were actionable G12C mutations. Two patients were found to have both EGFR and ALK alterations, both of whom received ALK-targeted therapy for first-line. With regard to six EGFR alterations, five were exon 19 deletions, and one was an exon 21 L858R mutation. No exon 20 or atypical EGFR mutations were defined. Among patients with BRAF alteration, the actionable V600E mutation was not identified. Other potential targets, including MET, RET, and NTRK alterations, were not detected.

### 3.5. First-Line Treatments

**Figure 3:**
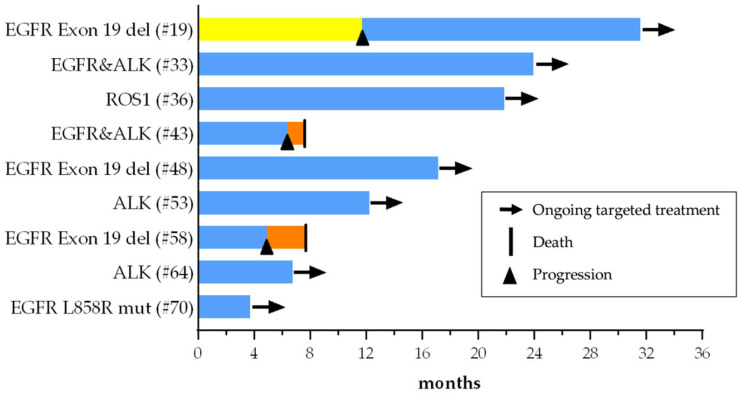
Swimmer plot of targeted therapies among patients with a potentially targetable alteration. Blue bars represent targeted therapies, yellow bar represents chemotherapy, and orange bars represent treatment-free follow-up periods. The numbers in parentheses indicate patients’ study IDs. del, deletion; mut, mutation.

There were 18 patients (25.7%) found to have a possible targeted therapy option for any treatment line; nine of them were able to receive this targetable treatment. Among these nine patients, eight received targeted treatment in the first line. The remaining patient with an EGFR exon 19 deletion was able to undergo targeted therapy following progression after six cycles of chemotherapy (Figure 3). Nine patients with a potential druggable alteration (six with KRAS G12C mutation and three with HER2 alteration) were not able to receive targeted therapy in any treatment line.

Considering the 62 patients without receiving first-line targeted therapy, 50 were treated with chemotherapy, and 7 with chemoimmunotherapy. Of the five remaining patients, two declined to receive the recommended therapy options and opted for treatment-free follow-up. The remaining three were unable to receive any treatment due to their performance status and comorbidities.

### 3.6. Impact of Targetable Alterations on Survival

The median follow-up period was 17.9 months (95% CI: 13.1–22.7), and the median OS of the entire patient cohort was 13 months.

**Figure 4:**
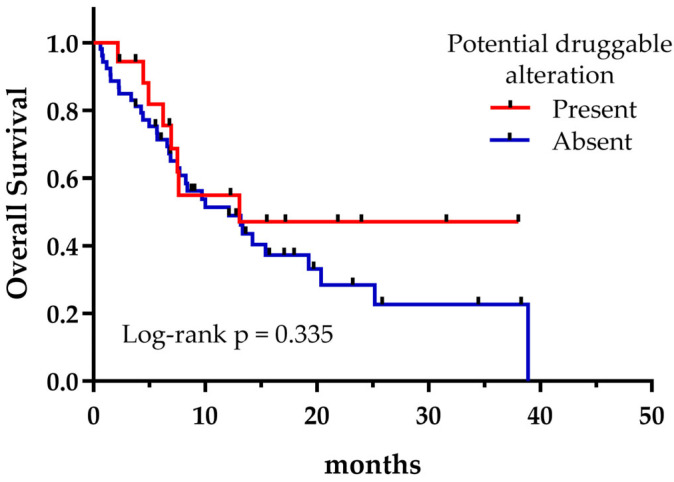
Overall survival regarding the presence or absence of potential targetable alterations.

**Figure 5:**
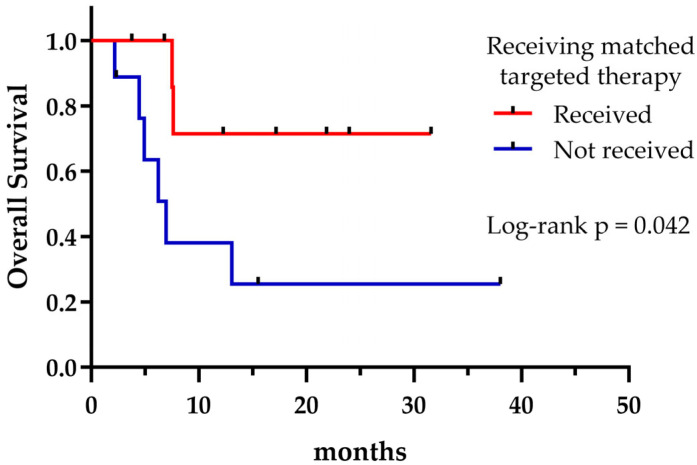
Overall survival according to receiving matched targeted therapy.

**Figure 6:**
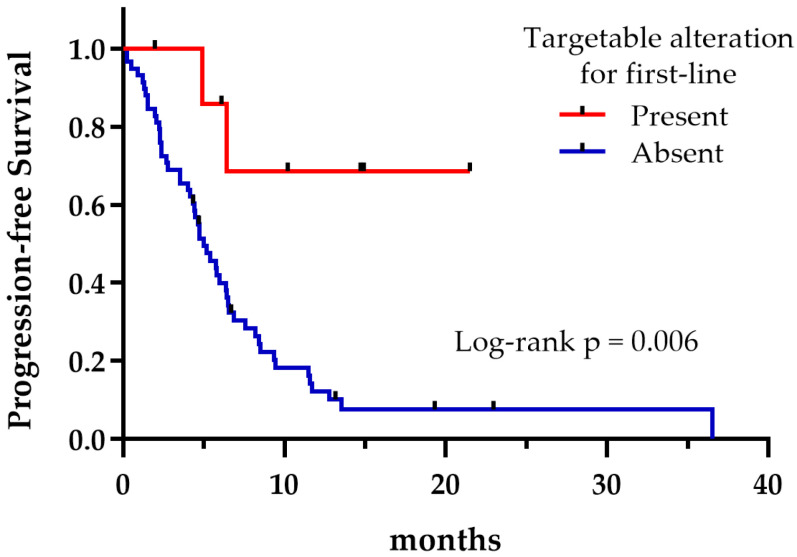
First-line progression-free survival regarding the presence or absence of targetable alterations.

Regardless of the treatment received, the presence of potentially druggable alterations was not associated with a significant effect on OS, as shown in Figure 4 (*p* = 0.335). However, patients harboring druggable alterations that could reach a matched targeted therapy at any treatment line exhibited a longer median OS compared to those who could not (not reached vs. 6.9 months, *p* = 0.042) (Figure 5). Not surprisingly, patients with a targetable alteration for first-line treatment demonstrated a significantly longer PFS compared to those without a targetable alteration for first-line (not reached vs. 4.9 months, *p* = 0.006) (Figure 6).

## 4. Discussion

In this study, we aimed to determine the molecular profile of our patients diagnosed with metastatic NSCLC and to emphasize the importance of achieving targeted treatment through molecular profiling. The findings of our study indicated that patients with a targetable alteration demonstrate a significant PFS and OS when the treatment is tailored based on that specific alteration.

It has become crucial to perform molecular testing to identify targetable alterations in patients with NSCLC, ensuring that patients receive the most appropriate treatment in the first-line setting [1,2,4]. NGS efficiently analyzes genomic changes using a limited sample size in a short duration, enhancing the likelihood of detecting targetable alterations [6]. However, many countries still face challenges in accessing NGS [11,15]. Therefore, the conventional tests covered by reimbursement of SSI still retain their importance [8]. Considering all of these conditions, we analyzed the results of NGS in combination with the results of conventional methods.

The median age at diagnosis and the female-to-male ratio were consistent with previous research [16,17,18]. We observed that the rate of female patients and the percentage of never smokers were comparable to a study investigating genetic mutations in patients with NSCLC in Türkiye [19]. The results of the present study also confirmed previously reported associations of EGFR and ALK with smoking history and gender, as these alterations are known to be more prevalent in female patients and in never smokers [19,20,21,22]. We could postulate that in order to find targetable alterations, female gender and never smokers could be prioritized for NGS as a cost-effective use of resources in Türkiye.

In line with previous studies conducted in Western populations, the top three molecular alterations in our cohort were the TP53, followed by KRAS and EGFR [23,24,25]. KRAS alteration was reported to be more prevalent in Western countries than in Asia [26]. We found KRAS alterations in 20% of the cases, similar to previous findings in Turkish patients with NSCLC [13,27]. In addition, we detected the targetable G12C mutation in approximately 40% of the KRAS alterations, as previously documented [28]. The relatively high prevalence of the G12C mutation suggests a potential opportunity for targeted therapy with G12C inhibitors, which could enhance patient outcomes in our population. Nevertheless, the limited accessibility of these treatment options poses a significant challenge. KRAS is among the most frequently reported alterations associated with a history of smoking and male gender [2,29]. However, we could not statistically observe this relationship in our study, possibly due to the relatively small number of patients.

The EGFR mutation rate varies across geographic regions, with the highest prevalence reported in East Asians (up to 78%) [30]. The prevalence in Türkiye has been found to be similar to non-Asian populations (10–16%) [13]. We detected EGFR mutations at a lower rate than reported in previous Turkish studies. Potential explanations for the observed discrepancy in the EGFR rate in our study include demographic and geographic differences, the EGFR variant found, tumor source (primary or metastatic), and the testing method performed.

The prevalence of ALK and ROS1 alterations in the present study was consistent with the national and global data reported [13,30,31]. We were unable to detect other targetable alterations, MET, BRAF V600E, RET, and NTRK, probably due to the limited number of patients in the study.

The findings across different studies highlight a significant disparity in the percentage of potentially actionable alterations. While the Korean study by Kim et al. reported a high rate of 75.2% [32], an Indian study showed a slightly lower but substantial rate of 64.8% [33]. In contrast, studies focusing on Western populations exhibited much lower rates, with only 16.5% to 36.4% of patients identified as harboring druggable alterations [24,25,34]. The differences in genetic backgrounds and the higher rates of EGFR mutations in the Eastern population might be the main reason for this geographic difference. A limited number of studies have been conducted in Türkiye; however, most of these have not evaluated all of the targetable alterations recommended by the current guidelines [18,19,35]. Although a direct comparison with Turkish studies is not feasible, the rate of 25.7% found in our study is comparable to rates reported in European studies. Our findings may suggest that the genetic landscape in Türkiye could share similarities with Western populations, which has implications for both diagnostic and therapeutic strategies. Further research focused on all targetable alterations is essential to enhance personalized medicine approaches in our region.

Real-world studies have shown that patients with NSCLC receiving at least one line of matched targeted therapy based on biomarker testing experienced prolonged survival [24,36]. Shah et al. reported that driver mutation-positive patients receiving targeted therapy had longer survival compared to those without targeted therapy, with a median OS of 26.7 vs. 9.3 months [33]. Simarro et al. revealed similar survival findings (26.2 vs. 8.8 months) [24]. In addition, they have been documented that patients being treated with targeted therapy exhibited a significantly longer first-line PFS when compared to those undergoing chemotherapy (13.4 vs. 5.2 months) [24,33]. In line with these findings, we found that patients with targetable alterations experienced longer survival times when they underwent matched targeted therapy. Patients in our study did not reach the median survival time when they received targeted therapy as their first-line treatment. On the other hand, patients without targeted therapy, most of whom received chemotherapy, exhibited poor survival times, consistent with the previously mentioned studies. The identification of actionable alterations through molecular profiling not only ensures the proper selection of targeted treatment but also precludes the use of immunotherapy in the first-line setting, which is almost ineffective in EGFR- and ALK-positive patients [2,37]. Accordingly, we observed that patients with EGFR, ALK, and ROS1 alterations received targeted therapy in the first-line setting.

It should be noted that the present study is subject to several limitations. First, the research was conducted on a limited number of patients. Consequently, it is imperative to be cautious when generalizing the results. Second, given that this was not a randomized study, baseline characteristics and treatment decisions may have differed across patients. Third, patients without targeted treatment options often failed to access immunotherapy in the first-line setting since reimbursement of immunotherapy in NSCLC in Türkiye covers only patients who have progressed on first-line chemotherapy. This could affect the survival rates we reported. Despite these limitations, we observed consistent results with the abovementioned molecular profiling studies.

## 5. Conclusions

The survival analysis in this study demonstrated the impact of the use of targeted therapies on the survival outcomes. Conducting molecular testing for all potential targetable alterations in NSCLC is the cornerstone of the treatment decision process and the key to access to new treatment options. Nevertheless, it is crucial to take into account the reimbursement policies and healthcare systems of each country, which can differ considerably.

## Figures and Tables

**Table 1:** Baseline characteristics of the patients (*n* = 70).

Variables	
Median age, years (range)	65 (43–78)
Gender, *n* (%)	
Female	16 (22.9)
Male	54 (77.1)
Smoking status, *n* (%)	
Never	15 (21.4)
Active or former	55 (78.6)
Smoking pack/years, median (IQR)	40 (31)
Histology, *n* (%)	
Adenocarcinoma	44 (62.9)
Squamous	22 (31.4)
Large-cell	4 (5.7)
PD-L1 TPS, *n* (%)	
<1%	35 (50)
≥1%	35 (50)
Number of metastatic sites, *n* (%)	
1	33 (47.1)
2	25 (35.7)
≥3	12 (17.2)
Liver metastasis, *n* (%)	
Yes	10 (14.3)
No	60 (85.7)
Bone metastasis, *n* (%)	
Yes	32 (45.7)
No	38 (54.3)
CNS metastasis, *n* (%)	
Yes	17 (24.3)
No	53 (75.7)

IQR, interquartile range; PD-L1, programmed death ligand 1; TPS, tumor proportion score; CNS, central nervous system.

**Table 2:** Molecular alterations with a frequency of less than 4% in all cohorts.

Alteration	*n* (%)
BRAF	2 (2.9)
KEAP1	2 (2.9)
ATM	2 (2.9)
PTEN	2 (2.9)
NOTCH4	2 (2.9)
SETD2	2 (2.9)
TSC1	2 (2.9)
ROS1	1 (1.4)
AKT1	1 (1.4)
ARID1A	1 (1.4)
BRCA1-2	1 (1.4)
MAP2K1	1 (1.4)
SMARCA4	1 (1.4)
FGFR1	1 (1.4)

## Data Availability

The data presented in this study are available in this article. Further inquiries can be directed to the corresponding author.

## References

[1] Li M.S.C., Mok K.K.S., Mok T.S.K. (2023). Developments in targeted therapy & immunotherapy—How non-small cell lung cancer management will change in the next decade: A narrative review. Ann. Transl. Med..

[2] Tan A.C., Tan D.S.W. (2022). Targeted Therapies for Lung Cancer Patients with Oncogenic Driver Molecular Alterations. J. Clin. Oncol..

[3] Riely G.J., Wood D.E., Ettinger D.S., Aisner D.L., Akerley W., Bauman J.R., Bharat A., Bruno D.S., Chang J.Y., Chirieac L.R. (2024). Non-Small Cell Lung Cancer, Version 4.2024, NCCN Clinical Practice Guidelines in Oncology. J. Natl. Compr. Cancer Netw..

[4] Hendriks L.E., Kerr K.M., Menis J., Mok T.S., Nestle U., Passaro A., Peters S., Planchard D., Smit E.F., Solomon B.J. (2023). Oncogene-addicted metastatic non-small-cell lung cancer: ESMO Clinical Practice Guideline for diagnosis, treatment and follow-up. Ann. Oncol..

[5] El-Deiry W.S., Goldberg R.M., Lenz H.-J., Shields A.F., Gibney G.T., Tan A.R., Brown J., Eisenberg B., Heath E.I., Phuphanich S. (2019). The current state of molecular testing in the treatment of patients with solid tumors, 2019. CA Cancer J. Clin..

[6] Moorcraft S.Y., Gonzalez D., Walker B.A. (2015). Understanding next generation sequencing in oncology: A guide for oncologists. Crit. Rev. Oncol./Hematol..

[7] Lazzari C., Bulotta A., Cangi M.G., Bucci G., Pecciarini L., Bonfiglio S., Lorusso V., Ippati S., Arrigoni G., Grassini G. (2020). Next Generation Sequencing in Non-Small Cell Lung Cancer: Pitfalls and Opportunities. Diagnostics.

[8] Tsao M.S., Yatabe Y. (2019). Old Soldiers Never Die: Is There Still a Role for Immunohistochemistry in the Era of Next-Generation Sequencing Panel Testing?. J. Thorac. Oncol..

[9] Laes J.-F., Aftimos P., Barthelemy P., Bellmunt J., Berchem G., Camps C., de Las Peñas R., Finzel A., García-Foncillas J., Hervonen P. (2018). The clinical impact of using complex molecular profiling strategies in routine oncology practice. Oncotarget.

[10] Pecciarini L., Brunetto E., Grassini G., De Pascali V., Ogliari F.R., Talarico A., Marra G., Magliacane G., Redegalli M., Arrigoni G. (2023). Gene Fusion Detection in NSCLC Routine Clinical Practice: Targeted-NGS or FISH?. Cells.

[11] Smeltzer M.P., Wynes M.W., Lantuejoul S., Soo R., Ramalingam S.S., Varella-Garcia M., Meadows Taylor M., Richeimer K., Wood K., Howell K.E. (2020). The International Association for the Study of Lung Cancer Global Survey on Molecular Testing in Lung Cancer. J. Thorac. Oncol..

[12] Bray F., Laversanne M., Sung H., Ferlay J., Siegel R.L., Soerjomataram I., Jemal A. (2024). Global cancer statistics 2022: GLOBOCAN estimates of incidence and mortality worldwide for 36 cancers in 185 countries. CA Cancer J. Clin..

[13] Cangır A.K., Yumuk P.F., Sak S.D., Akyürek S., Eralp Y., Yılmaz Ü., Selek U., Eroğlu A., Tatlı A.M., Dinçbaş F.Ö. (2022). Lung Cancer in Turkey. J. Thorac. Oncol..

[14] Pennell N.A., Arcila M.E., Gandara D.R., West H. (2019). Biomarker Testing for Patients with Advanced Non-Small Cell Lung Cancer: Real-World Issues and Tough Choices. American Society of Clinical Oncology Educational Book.

[15] Ferreira-Gonzalez A., Ko G., Fusco N., Stewart F., Kistler K., Appukkuttan S., Hocum B., Allen S.M., Babajanyan S. (2024). Barriers and facilitators to next-generation sequencing use in United States oncology settings: A systematic review. Future Oncol..

[16] Kızılırmak D., Yılmaz Kaya Z., Gökçimen G., Havlucu Y., Cengiz Özyurt B., Gündoğuş B., Esendağlı D., Serez Kaya B., Yılmam İ., Aydemir Y. (2023). Lung cancer from suspicion to treatment: An indicator of healthcare access in Turkey. Cancer Epidemiol..

[17] Zahed H., Feng X., Sheikh M., Bray F., Ferlay J., Ginsburg O., Shiels M.S., Robbins H.A. (2024). Age at diagnosis for lung, colon, breast and prostate cancers: An international comparative study. Int. J. Cancer.

[18] Demiray A., Yaren A., Karagenç N., Bir F., Demiray A.G., Er K., Tokgün O., Elmas L., Akça H. (2018). The Frequency of EGFR and KRAS Mutations in the Turkish Population with Non-small Cell Lung Cancer and their Response to Erlotinib Therapy. Balk. J. Med. Genet..

[19] Özçelik N., Aksel N., Bülbül Y., Erdoğan Y., Güldaval F., Gül Ş.K., Bircan A., Can A., Öz N., Şentürk A. (2019). Regional distribution of genetic mutation in lung cancer in Turkey (REDIGMA). Tuberk Toraks.

[20] Chapman A.M., Sun K.Y., Ruestow P., Cowan D.M., Madl A.K. (2016). Lung cancer mutation profile of EGFR, ALK, and KRAS: Meta-analysis and comparison of never and ever smokers. Lung Cancer.

[21] Zhang Y.-L., Yuan J.-Q., Wang K.-F., Fu X.-H., Han X.-R., Threapleton D., Yang Z.-Y., Mao C., Tang J.-L. (2016). The prevalence of EGFR mutation in patients with non-small cell lung cancer: A systematic review and meta-analysis. Oncotarget.

[22] Zhu Q., Zhan P., Zhang X., Lv T., Song Y. (2015). Clinicopathologic characteristics of patients with ROS1 fusion gene in non-small cell lung cancer: A meta-analysis. Transl. Lung Cancer Res..

[23] Tsoulos N., Papadopoulou E., Metaxa-Mariatou V., Tsaousis G., Efstathiadou C., Tounta G., Scapeti A., Bourkoula E., Zarogoulidis P., Pentheroudakis G. (2017). Tumor molecular profiling of NSCLC patients using next generation sequencing. Oncol. Rep..

[24] Simarro J., Pérez-Simó G., Mancheño N., Ansotegui E., Muñoz-Núñez C.F., Gómez-Codina J., Juan Ó., Palanca S. (2023). Impact of Molecular Testing Using Next-Generation Sequencing in the Clinical Management of Patients with Non-Small Cell Lung Cancer in a Public Healthcare Hospital. Cancers.

[25] Kuang S., Fung A.S., Perdrizet K.A., Chen K., Li J.J.N., Le L.W., Cabanero M., Karsaneh O.A.A., Tsao M.S., Morganstein J. (2022). Upfront Next Generation Sequencing in Non-Small Cell Lung Cancer. Curr. Oncol..

[26] Izumi M., Suzumura T., Ogawa K., Matsumoto Y., Sawa K., Yoshimoto N., Tani Y., Watanabe T., Kaneda H., Mitsuoka S. (2019). Differences in molecular epidemiology of lung cancer among ethnicities (Asian vs. Caucasian). J. Thorac. Dis..

[27] Eser M., Hekimoglu G., Yarar M.H., Canbek S., Ozcelik M. (2024). KRAS G12C mutation in NSCLC in a small genetic center: Insights into sotorasib therapy response potential. Sci. Rep..

[28] Karachaliou A., Kotteas E., Fiste O., Syrigos K. (2024). Emerging Therapies in Kirsten Rat Sarcoma Virus (+) Non-Small-Cell Lung Cancer. Cancers.

[29] Kahraman Çetin N., Erdoğdu İ.H., Bozkurt E., Meteoğlu İ. (2021). Evaluation of the Mutation Profile via Next-Generation Sequencing in a Turkish Population with Non-small Cell Lung Cancer. Balk. Med. J..

[30] Fois S.S., Paliogiannis P., Zinellu A., Fois A.G., Cossu A., Palmieri G. (2021). Molecular Epidemiology of the Main Druggable Genetic Alterations in Non-Small Cell Lung Cancer. Int. J. Mol. Sci..

[31] Lin J.J., Riely G.J., Shaw A.T. (2017). Targeting ALK: Precision Medicine Takes on Drug Resistance. Cancer Discov..

[32] Kim J.H., Yoon S., Lee D.H., Jang S.J., Chun S.-M., Kim S.-W. (2021). Real-world utility of next-generation sequencing for targeted gene analysis and its application to treatment in lung adenocarcinoma. Cancer Med..

[33] Shah M., Noronha V., Patil V., Singh A.K., Menon N., Goud S., Shah S., More S., Kapoor A., Mishra B.K. Genomic Profiling of Driver Gene Alterations in Patients with Non-Small Cell Lung Cancer, Patterns of Treatment and Impact on Survival Outcomes: A Single Center Experience of More Than 1200 Patients. Clin. Lung Cancer.

[34] Porta C., Pradelli L., Sicari E., Castellani S., Sivakumar S., Sokol E., Montesion M., Wieland T., Rambichler J., Minari R. (2023). Liquid biopsy comprehensive genomic profiling of lung cancer in the Italian population: A real-world experience. Lung Cancer.

[35] Gün E., Çakır İ.E., Ersöz H., Oflazoğlu U., Sertoğullarından B. (2024). The Epidermal Growth Factor, Anaplastic Lymphoma Kinase, and ROS Proto-oncogene 1 Mutation Profile of Non-Small Cell Lung Carcinomas in the Turkish Population: A Single-Center Analysis. Thorac. Res. Pract..

[36] Bhandari N.R., Hess L.M., He D., Peterson P. (2023). Biomarker Testing, Treatment, and Outcomes in Patients With Advanced/Metastatic Non-Small Cell Lung Cancer Using a Real-World Database. J. Natl. Compr. Cancer Netw..

[37] Guaitoli G., Tiseo M., Di Maio M., Friboulet L., Facchinetti F. (2021). Immune checkpoint inhibitors in oncogene-addicted non-small cell lung cancer: A systematic review and meta-analysis. Transl. Lung Cancer Res..

